# Improved functionalization of oleic acid-coated iron oxide nanoparticles for biomedical applications

**DOI:** 10.1007/s11051-012-1100-5

**Published:** 2012-08-07

**Authors:** Maarten Bloemen, Ward Brullot, Tai Thien Luong, Nick Geukens, Ann Gils, Thierry Verbiest

**Affiliations:** 1 Department of Chemistry, KU Leuven, Celestijnenlaan 200D, Box 2425, 3001 Heverlee (Leuven), Belgium; 2 PharmAbs, The KU Leuven Antibody Center, KU Leuven, O&N II, Herestraat 49, Box 824, 3000 Leuven, Belgium; 3 Faculty of Pharmaceutical Sciences, KU Leuven, O&N II Herestraat 49, Box 824, 3000 Leuven, Belgium

**Keywords:** Oleic acid-coated nanoparticles, Superparamagnetic, Surface modification, Silane, Colloidal stability, Iron oxide

## Abstract

Superparamagnetic iron oxide nanoparticles can provide multiple benefits for biomedical applications in aqueous environments such as magnetic separation or magnetic resonance imaging. To increase the colloidal stability and allow subsequent reactions, the introduction of hydrophilic functional groups onto the particles’ surface is essential. During this process, the original coating is exchanged by preferably covalently bonded ligands such as trialkoxysilanes. The duration of the silane exchange reaction, which commonly takes more than 24 h, is an important drawback for this approach. In this paper, we present a novel method, which introduces ultrasonication as an energy source to dramatically accelerate this process, resulting in high-quality water-dispersible nanoparticles around 10 nm in size. To prove the generic character, different functional groups were introduced on the surface including polyethylene glycol chains, carboxylic acid, amine, and thiol groups. Their colloidal stability in various aqueous buffer solutions as well as human plasma and serum was investigated to allow implementation in biomedical and sensing applications.

**Electronic supplementary material:**

The online version of this article (doi:10.1007/s11051-012-1100-5) contains supplementary material, which is available to authorized users.

## Introduction

For many years, iron oxide nanoparticles have been the subject of intensive research. These cost-effective and non-toxic particles are used nowadays in many applications such as magnetic storage, drug delivery, biosensing, magnetic separation, and contrast reagents for imaging techniques (Laurent et al. 2008; Speliotis 1999). A common method to make such particles is coprecipitation where iron(II+) and (III+) ions are dissolved in water and precipitated using ammonia or sodium hydroxide (Sun et al. 2006). Drawbacks are the poor monodispersity and irregular shape of these particles. Recently, multiple methods have been published to synthesize monodisperse nanoparticles in organic solvents. Sun et al. (2004) made high-quality magnetite nanocrystals with small size distribution by thermal decomposition of iron(III) acetylacetonate in phenyl ether. A similar method reported by Park et al. (2004) uses iron-oleate as a precursor and oleic acid as a capping agent. This results in nanoparticles with a hydrophobic coating since the polar end groups are attached to the surface. Capping agents such as oleic acid are often used because they form a protective monolayer, which is strongly bonded. This is necessary for making monodisperse and highly uniform nanoparticles (Bronstein et al. 2007; Zhang et al. 2006). For biomedical applications in aqueous environments, this hydrophobic coating has to be replaced with a hydrophilic coating. The so-called ligand exchange is well known for noble metal nanoparticles where, for instance, thiol groups attach strongly to the surface, thereby forming monolayers by self-assembly. A similar approach is possible for iron oxide nanoparticles using polymers or α-cyclodextrin (Fauconnier et al. 1997; Wang et al. 2003; Park et al. 2011). Since these layers are often not covalently bonded to the surface, high ionic strength or extreme pH conditions might alter their interaction. An elegant alternative is silane chemistry, which is based on the reactivity of silanol molecules, formed by the hydrolyzation of alkoxy silane (Soderholm and Shang 1993; Zhang et al. 2002). A high degree of control and reproducibility is possible when the appropriate reaction conditions are chosen. The possibility to introduce a large variety of functional groups onto the surface of magnetic nanoparticles makes this approach very valuable. Other advantages are the high stability and density of the formed silicon oxide layer. However, introducing silane molecules onto the surface of oleic acid-stabilized nanoparticles has only been scarcely reported so far. De Palma et al. (2007) described a method which uses hexane as a solvent and acetic acid as a catalyst to form the reactive silanol molecules. Larsen et al. (2009) published a protocol with toluene as the solvent and water being the catalyst; triethylamine was added to facilitate the reaction. Kohler et al. (2004) also managed to introduce silanes, but pre-treated the oleic acid coating with a mixture of 1 M ammonium hydroxide in 1-butanol. These methods have serious drawbacks, e.g., the extensive reaction time (24–72 h) or pre-treatment procedure.

In this paper, we present an improved nanoparticles functionalization method. The obtained superparamagnetic nanoparticles are thoroughly characterized by electron microscopy, X-ray powder diffraction, and vibrating sample magnetometry. Infrared spectroscopy is used to confirm the presence of the functional groups on the surface after reaction with silane molecules. By performing this reaction in an ultrasonication bath, the reaction time is greatly reduced, while avoiding crosslinking and thus maintaining the monodispersity. The colloidal stability of the resulting nanoparticles was extensively tested in different aqueous media at several pHs as well as in human serum and plasma, which demonstrates their applicability in biomedical applications. A large variety of functional groups were introduced to the surface, proving the generic character of the method.

## Materials and methods

### Materials

Sodium oleate and iron(III) chloride hexahydrate (97 %) were obtained from Sigma Aldrich, ethanol (absolute) and oleic acid from VWR, and heptane and toluene from Fisher Scientific. Triethyl amine was ordered at Janssen Chimica. Acetone was purchased at Chem Lab. Methoxy(polyethyleneoxy)propyltrimethoxysilane (90 %, 9–12 PE-units), 3-mercaptopropyltrimethoxysilane (95 %), *N*-(trimethoxysilylpropyl) ethylenediamine triacetic acid trisodium salt (45 %), and 3-Aminopropyltrimethoxysilane (97 %) were obtained from ABCR. 1-Octadecene (90 %, technical grade) was purchased at Acros.

### Synthesis of nanoparticles

Superparamagnetic iron oxide nanoparticles were prepared by the method published by Park et al. (2004) with minor modifications. It consists of two separate reactions, first preparing an iron-oleate precursor, which is later transformed into iron oxide nanocrystals.

For the synthesis of the precursor, 36.5 g (120 mmol) sodium oleate and 10.8 g (40 mmol) iron(III) chloride hexahydrate were dissolved in a mixture of 80 mL ethanol, 60 mL MilliQ water, and 140 mL heptane. This mixture was heated to reflux at 70 °C for 4 h under an argon atmosphere. Afterward, the upper heptane layer, which contains the iron-oleate, was separated using a separatory funnel and washed three times with 40 mL MilliQ water. As a final step, the heptane was evaporated using a rotavapor, resulting in a dark brown waxy solid.

The iron oxide nanoparticles' synthesis starts with mixing 36 g (40 mmol) of the iron-oleate with 5.7 g (20 mmol) of oleic acid and 200 g of 1-octadecene in a 500 mL three-neck flask. This mixture was first heated to 100 °C for 5 min to evaporate all remaining heptane. After fitting a reflux cooler, the mixture was heated further to 320 °C and kept at that temperature for 30 min. Around 250 °C, the decarboxylation of the oleate starts, producing a large amount of CO_2_ gas. Afterward, the reaction mixture is cooled down to room temperature by removing the heat source. 500 mL of ethanol is added to precipitate the freshly prepared nanoparticles. Separation was done by centrifugation, after which the particles were washed three times with ethanol. After drying, the nanocrystals were dispersed in heptane (with one drop of oleic acid) in high concentration (100 mg/mL) for long-term storage.

### Functionalization

The new protocol presented here was partially based on a method published by Larsen et al. (2009), but with important modifications. In a typical functionalization experiment, 100 mg of iron oxide nanoparticles (in heptane, stock solution) were mixed with 50 mL of toluene. To this mixture, 2.5 mL of triethylamine, 0.05 mL of MilliQ water, and 0.5 mL of the desired silane were added. The beaker was then placed in an ultrasonication bath for 5 h. The temperature of the water inside this bath was kept at 50 °C during the reaction. Afterward, the volume of the reaction mixture was doubled by adding heptane to the solution, 50 mL in this case. The mixture was placed on a magnet to precipitate the functionalized nanoparticles. The supernatant was decanted and the particles were washed three times with acetone and precipitated by a magnet. After drying under reduced pressure for 15 min, the sample was weighed and dissolved in MilliQ water or the appropriate medium.

### Equipment and characterization

The ultrasonication bath used in the particle functionalization was a Bransonic Model 5510 sonicator with a capacity of 10 L. The built-in heating was never used.

Transmission electron microscopy measurements were performed on a 80 kV Zeiss EM-900 using 300 mesh Formvar coated copper grids. Distribution data were calculated by ImageJ. Oleic acid-coated nanoparticles were dispersed in heptane and deposited onto the grid.

Fourier transform infrared spectra were obtained using a Bruker Alpha FT-IR spectrometer equipped with a Platinum ATR module.

Dynamic light scattering and zeta potentials were measured on a Brookhaven 90plus particle analyzer. The internal detector was positioned at 90°.

UV–Vis Spectrometry was performed on a Perkin Elmer Lambda 900 spectrometer.

Vibrating sample magnetometry experiments were conducted on a VSM Maglab setup from Oxford Instruments.

X-ray powder diffraction spectra were obtained in reflection (Bragg–Brentano geometry) using a Rigaku Rotaflex diffractometer fitted with a Rigaku RU-200B rotating Cu-anode (λ = 1.54 Å) at a power of 4 kW. The diffracted X-rays were collected after Ni-filtering on a scintillation counter. Samples were deposited on a glass microscope slide from solution.

Samples, to test the colloidal stability, were prepared by adding concentrated nanoparticle dispersion (in water) to the appropriate medium. The protocol for collection of human plasma and serum is described in Online Resource 1. Absorbance values were measured at a wavelength of 1,000 nm.

## Results and discussion

**Fig. 1:**
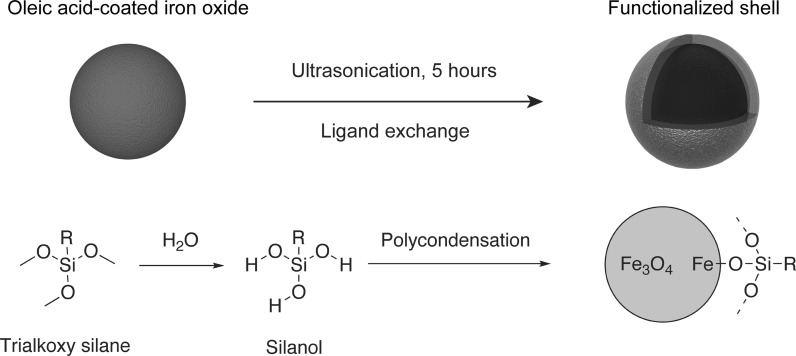
Overview of the chemical reactions during the functionalization of iron oxide nanoparticles with silanes. The formation of the silanol molecule occurs by reaction with water. Subsequent polycondensation renders a silane network on the surface of the nanoparticle

Efficient surface modification of superparamagnetic nanoparticles is crucial for their application in biomedicine. Recent advances in high temperature syntheses already improved the shape and monodispersity of the core. As mentioned before, these particles are only soluble in apolar solvents because of their oleic acid coating. To change the polarity of the layer to being hydrophilic, a ligand exchange is essential. We preferred interaction with alkoxy silanes to polymers because of the covalent bond formation, resulting in more versatile and robust nanoparticles. The reaction mechanism is shown in Fig. 1. After formation of the active silanol molecule, it reacts with the surface OH groups of the iron oxide nanoparticle. This results in the formation of a Fe–O–Si bond. Metal oxides are known to have reactive hydroxyl groups present on their surface, caused by the adsorption of water (Cornell and Schwertmann 2003). This is similar to functionalization of silicon oxide substrates (Fadeev and McCarthy 2000). The density and structure of the shell depends largely on the reaction conditions, being a combination of linear polymerization, network formation, and covalent attachment to the iron oxide surface. Although chlorosilanes would react faster than alkoxy silanes, we opted for the latter. Chlorosilanes release hydrogen chloride upon reaction, which is incompatible with iron oxide. On top of that, their reactivity is often too high to allow sufficient control of the reaction.

**Fig. 2:**
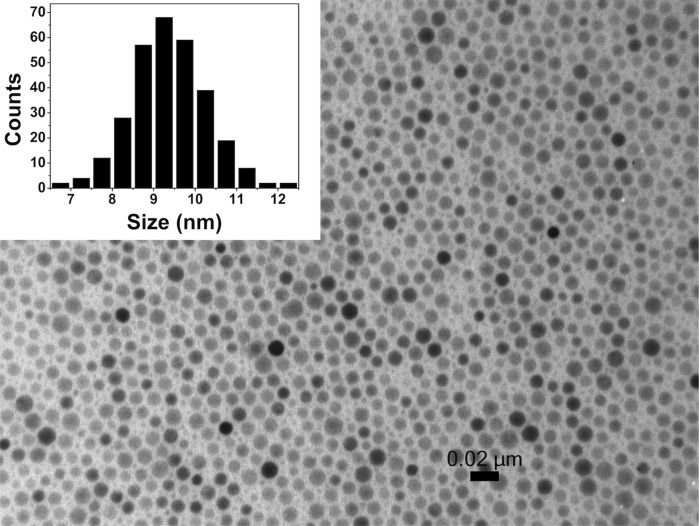
Transmission electron microscopy image of oleic acid-stabilized iron oxide nanoparticles. The *scale bar* represents 20 nm. The *inset* shows the size distribution: 9.3 ± 1.6 nm

The nanoparticle synthesis published by Park et al. (2004) allowed us to make high-quality spherical nanoparticles at large scale. Figure 2 shows a transmission electron microscopy (TEM) image of the oleic acid-coated nanoparticles. As can be seen in the inset, the nanoparticle distribution is narrow. A mean size of 9.3 nm was calculated by a Gaussian fit with a spread of ±1.6 nm (one sigma).

**Fig. 3:**
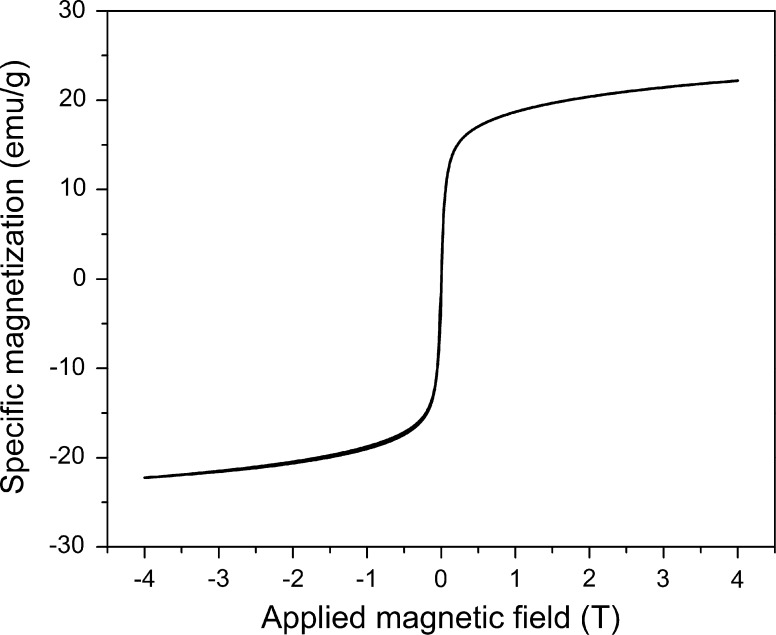
Vibrating sample magnetometry signal of the nanoparticles showing a hysteresis curve. The data points are fitted by a Langevin function to determine the magnetic core size

Since superparamagnetism is an important property of the nanoparticles, vibrating sample magnetometry (VSM) measurements were performed. Figure 3 shows the data of the oleic acid-coated particles. The applied field was varied between 4 and −4 Tesla, while recording the remnant magnetization. No coercivity or magnetic remanence is observed, which is typical for superparamagnetic nanoparticles. The hysteresis loop can be fitted by a Langevin function to deduct the size of the magnetic core. In this case, a core diameter of 10.53 nm was obtained. This value is comparable to the size determined by TEM measurements.

**Fig. 4:**
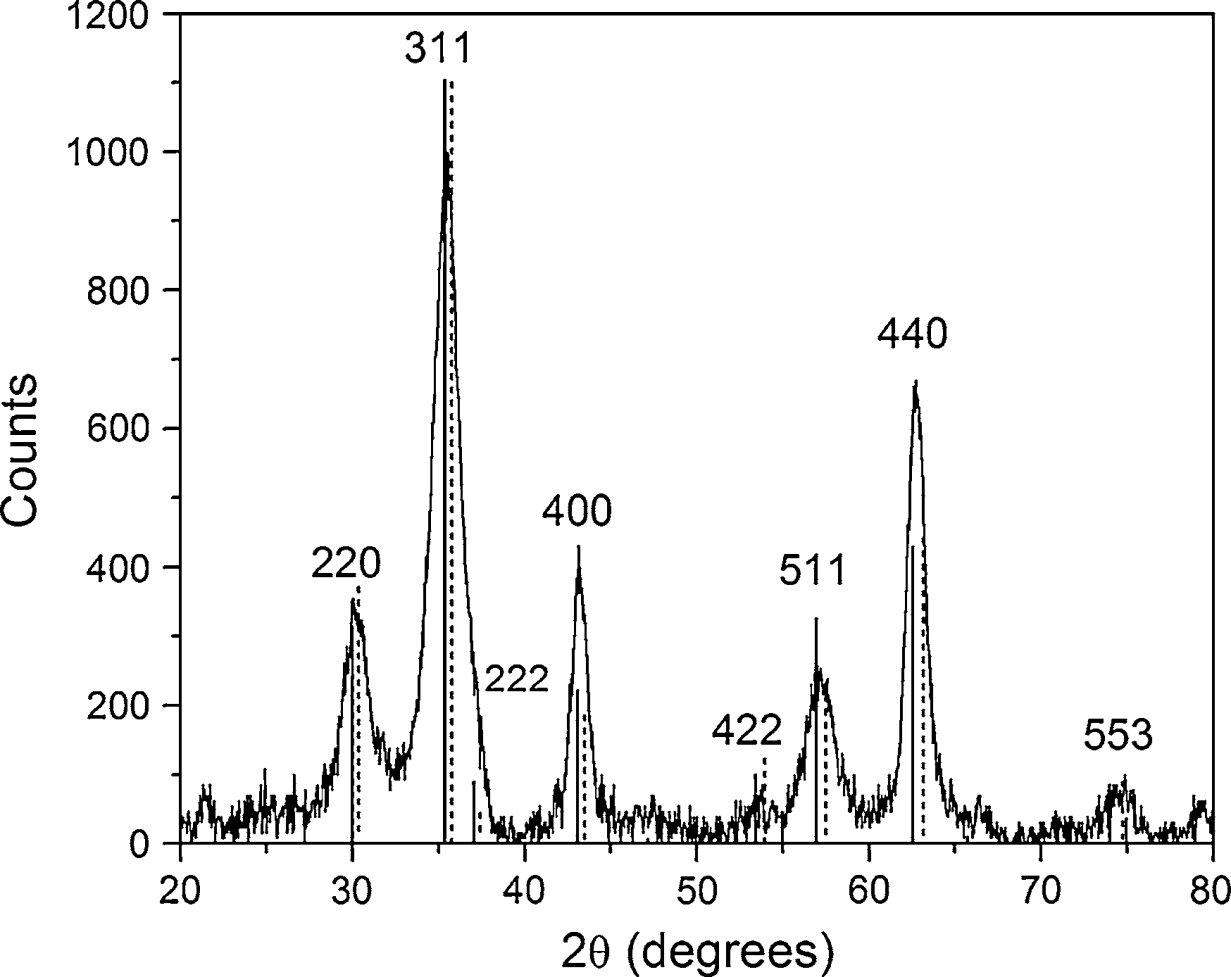
X-ray powder diffraction spectrum of the oleic acid-coated iron oxide nanoparticles. The *solid and dashed vertical drop-down lines* represent the peak positions of a reference magnetite and maghemite spectrum, respectively (AMCSD 0007824 and 0007899)

The crystal structure of the oleic acid-coated iron oxide nanoparticles was determined via X-ray powder diffraction (step size = 0.05°, dwell time = 40 s). As Fig. 4 indicates, the spectrum closely resembles the reference spectrum of magnetite. However, since the difference between maghemite and magnetite is very subtle in X-ray diffraction, no conclusions about the exact composition can be drawn. Therefore, we can only state that the crystals produced in the synthesis are superparamagnetic iron oxide nanoparticles, consisting of magnetite, maghemite, or a mixture of both. By means of the Scherrer equation, the crystal size can be derived from the peak broadening in the spectrum (Brullot et al. 2012). By means of MDI Jade, the (400) peak was fitted with a pseudo-Voight function after polynomial background subtraction, resulting in a size of 9.3 ± 0.7 nm. This value corresponds very well with the size determined by the TEM measurements.

Compared to previously reported functionalization methods, the method presented here has several advantages. (1) The reaction time is reduced to 5 h compared to 24 or 72 h as described by Larsen et al. (2009) and De Palma et al. (2007). (2) Using a sonicator, crosslinking of particles during the reaction is strongly reduced, thereby maintaining the monodispersity of the nanoparticles. In this case, the exact mechanism is unknown, but in general, sonication of a solution introduces microbubbles, which subsequently implode. These implosions generate high temperatures inside and around the cavity as well as a shock wave upon collapse. The surrounding liquid quickly disperses the heat, allowing the use of fragile organic materials (Mason and Lorimer 2002; Morel et al. 2008). (3) Another advantage is the elevated concentration of nanoparticles (2 mg/mL) during the synthesis, which reduces the amount of solvent needed.

The functionalization procedure was performed with four different trialkoxy silanes. Polyethylene glycol (PEG), carboxylic acid, amine, and thiol groups were introduced on the surface for various reasons. Thiol and amine groups are excellent anchor points for subsequent coating with a gold layer, e.g., by reduction of a gold containing salt. This is particularly useful in biomedical applications where the plasmonic response of gold is used to heat the environment or to release drugs at a specific location (Brullot et al. 2011; Janib et al. 2010). PEG chains on the surface, on the other hand, provide the nanoparticles with excellent dispersibility in water. These particles can be used to form magnetic fluids for applications like magnetic hyperthermia or thermo-ablation (Prasad et al. 2007). The introduction of carboxylic acid and amine groups can be utilized for bioconjugation of proteins to the nanoparticles. Typical chemical coupling reagents like EDC–NHS or glutaraldehyde link these functional groups to the target protein via an amide or imine bond. These bioconjugates are often used for detection and magnetic separation.

Successful modification of the nanoparticles’ surface was confirmed by Fourier transform infrared measurements (FTIR). Table 1 gives an overview of the vibrations measured on the oleic acid- and silane-coated particles. For the unmodified iron oxide cores, the characteristic bands of the asymmetric stretching, symmetric stretching, and scissoring of CH_2_ are visible at 2,919, 2,850, and 1,436 cm^−1^, respectively (Zhang et al. 2006; Chen et al. 2008a, b). The stretching of the C=O double bond is clearly shown by the peak at 1,709 cm^−1^. This was expected since the peak resembles free oleic acid, indicating that a significant amount of unbound surfactant is present, which can be related to the storage conditions. The presence of a peak at 1,462 cm^−1^, coming from in-plane OH bending, supports this idea. Zhang et al. (2006) reported that the C=O peak shifts to 1,541 and 1,639 cm^−1^ when the molecule is attached to a ferrite surface. The carboxylic acid groups are then present in a COO^−^ conformation. If the spectrum is magnified, these vibrations are also visible in our case at 1,541 and 1,635 cm^−1^, although they are fairly small. The wavenumber separation (of 94 cm^−1^) between those two peaks is an indication for how the oleate and the iron atoms on the surface interact. Because the difference is smaller than 110 cm^−1^, a chelating bidentate interaction can be derived from the spectrum. The iron oxide core itself shows a characteristic vibration at 598 cm^−1^, related to the Fe–O bonds (Zhang et al. 2006).

**Table 1:** Overview of the different vibrations related to the different coatings around the nanoparticle

Surface of Fe_3_O_4_	IR vibrations (cm^−1^)
Oleic acid	3,005 (HC=), 2,919 (CH_2_), 2,850 (CH_2_), 1,709 (C=0), 1,635 (COO^−^), 1,541 (COO^−^), 1,462 (OH), 1,436 (CH_2_), 598 (Fe–O)
COOH silane	3,600–3,000 (OH), 2,932 (CH_2_), 1,612 (COO^−^), 1,452 (CH_2_), 1,396 (COO^−^), 1,113 & 1,089 & 1,007 (Si–O), 585 (Fe–O)
PEG silane	3,600–3,000 (OH), 2,860 (PEG CH_2_), 1,643 (H_2_O), 1,454 & 1,349 & 1,297 & 1,250 & 1,047 & 947 (CH_2_–O–CH_2_), 1,198 (O–CH3), 620 (Fe–O)
NH_2_ silane	3,004 (OH & NH_2_), 2,922 (CH_2_), 2,850 (CH_3_), 1,543 (NH_3_^+^), 1,400 (CH_3_COOH), 1,224 (Si–C), 1,073 (Si–O–R), 773 (NH_2_), 617 (Fe–O)
SH silane	3,600–2,500 (OH & CH_2_), 2,600–2,550 (SH), 1,645 (H_2_O), 1,430 (CH_2_), 1,035 (Si–O), 590 (Fe–O)

The original spectra can be found in Online Resource 1

The presence of carboxylic acid groups, after functionalization, is proven by the vibrations at 1,612 and 1,396 cm^−1^. These correspond to the asymmetric and symmetric stretching of the COO^−^ group, respectively. Other important features are the 1,113, 1,089, and 1,007 cm^−1^ bands due to the stretching of the Si–O bond (Can et al. 2009; Ma et al. 2003).

The PEG silane shows distinct bands caused by the ether functions in the chain, to which several peaks between 1,454 and 947 cm^−1^ can be attributed. The high hydrophilicity of the PEG chain is expressed by the presence of a water peak in the spectrum. Even after extensive drying, this peak remains, indicating that the water is trapped by hydrogen bonding (De Palma et al. 2007). Several papers already reported that more information about the structure of the PEG layer on the nanoparticles surface can be derived from the FTIR spectrum. Compared to these reports, the conformation of the poly ethylene glycol layer is partially crystalline and partially amorphous. Small shoulders at 1,244, 1,460, and 1,470 cm^−1^ are present in the spectrum, indicating crystalline parts in the coating. On the other hand, the amorphous bands are visible at 948, 1,140, and 1,250 cm^−1^ (Harder et al. 1998; Valiokas et al. 1999).

For the amino silane-coated nanoparticles, the most important peaks are visible at 3,004, 1,543, and 773 cm^−1^ corresponding to the protonated amines. Because this protonation was done by adding acetic acid, a peak at 1,400 cm^−1^ appears. Mercapto silane-coated particles show a broad band above 2,500 cm^−1^, consisting of a combination of OH, CH_2_, and SH vibrations. Also, a small peak caused by the presence of water is visible. Typical peaks for Si–O en Fe–O also appear at 1,035 and 590 cm^−1^, respectively.

**Fig. 5:**
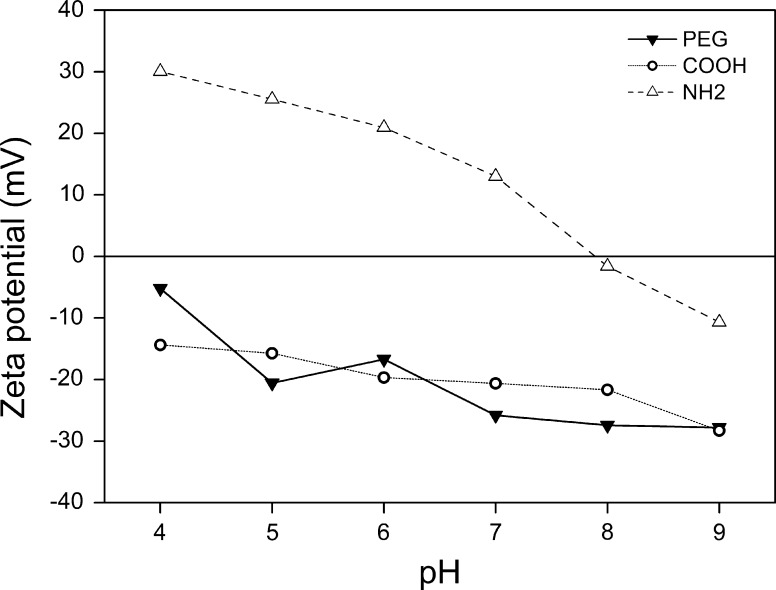
Zeta potential values for PEG-, carboxylic acid-, and amine-coated nanoparticles in various pH solutions. Every data point was derived from 10 measurements by the software

Further experiments were only conducted on the PEG, COOH, and amine coatings, since the thiol particles were only stable in water for a very short time, regardless of the pH. In general, the stability of nanoparticles dispersed in aqueous media can be expressed by the zeta potential. Theoretically, it refers to the potential difference between the slipping plane in the electronic double layer and the bulk potential. If the potential has an absolute value higher than 25–30 mV, it is generally accepted that the particles are electrostatically stable (Xu 2001). Although colloidal stability is related to electrostatic and steric repulsion, zeta potential measurements usually give a good indication. Figure 5 shows the combined data of the PEG-, carboxylic acid-, and amine-coated nanoparticles. A clear downward trend for the zeta potential of NH_2_ is visible when the pH increases. This can be related to the lowering of the surface charge due to deprotonation of the amine at high pH. A similar, but reverse, trend can be observed for COOH since the acid becomes protonated at low pH, thereby losing its negative charge. For PEG-coated particles, the zeta potential shows similar behavior even though polyethylene glycol chains have no pH responsive groups. The incorporation of ions into the PEG layer can explain this trend, taking into consideration that sodium hydroxide and hydrogen chloride were added to adjust the pH of the solution (Schweiss et al. 2001; Kreuzer et al. 2003). Nevertheless, the PEG-coated particles were stable over the entire pH range, indicating that their stability is caused by steric repulsion rather than electrostatic repulsion. For the COOH-coated nanoparticles, solutions of pH 4 and 5 showed extensive aggregation and consequent precipitation. A similar effect was observed for the amine-coated particles, this time for dispersions of pH 7 to 9. The profound impact of the pH on the stability of COOH and NH_2_ coatings shows that electrostatic repulsion is crucial for these types of dispersions (De Palma et al. 2007; Chen et al. 2008a, b).

To study the effect of buffer solutions on the colloidal stability, various dispersions with different salt concentrations were prepared. To our knowledge, no extensive studies have been conducted on magnetic nanoparticle dispersions in different buffer media at different pHs and concentrations (Barreto et al. 2011; Bhattacharya et al. 2011). Nevertheless, this information can be very valuable for subsequent reactions or applications of the nanoparticles. Five commonly used buffer reagents were chosen, covering the entire pH range between 4 and 9. These included sodium acetate, 2-(*N*-morpholino)-ethanesulfonic acid (MES), sodium hydrogen phosphate, 2-amino-2-hydroxymethyl-propane-1,3-diol (TRIS), and glycine. The concentration of the reagents ranged from 0.1 to 0.025 M, while the particle concentration was fixed at 0.25 mg/mL. Table 2 provides an overview of the stability in different buffer solutions, each having three different concentrations. Carboxylic acid-coated nanoparticles show excellent stability in different buffers above pH 4, which can be related to the presence of charged carboxylate groups on their surface, providing sufficient electrostatic repulsion. For the NH_2_-coated particles, the instability can be explained by the lack of charged functional groups. This is partially caused by the pH, which is too high for sufficient stabilization. On top of that, the high ionic strength and ion size largely influence the zeta potential. These results are in perfect agreement with the zeta potential measurements and the observed correlation between the presence of surface charges and colloidal stability. The particles with polyethylene glycol chains on their surface show good stability in phosphate, glycine, and TRIS-based buffer solutions. The instability in MES and acetate buffer, however, is somewhat unexpected since the zeta potential measurements showed that PEG provides mainly steric hindrance rather than electrostatic repulsion. Ion adsorption onto the coated surface is expected to be the cause of this instability.

**Table 2:** Overview of the stability of the nanoparticles in buffer solutions

Coating	CH_3_COOH/CH_3_COONa			CH_3_COOH/CH_3_COONa			MES/HCl		
100 %	pH4			pH5			pH6		
	0.1 M	0.05 M	0.025 M	0.1 M	0.05 M	0.025 M	0.1 M	0.05 M	0.025 M
NH_2_	+	+	+	+	+	+	±	±	+
PEG	+	+	+	±	±	±	±	±	±
COOH	±	+	+	+	+	+	+	+	+

Coating	NaH_2_PO_4_/Na_2_HPO4			TRIS/HCL			Glycine/NaOH		
100 %	pH7			pH8			pH9		
	0.1 M	0.05 M	0.025 M	0.1 M	0.05 M	0.025 M	0.1 M	0.05 M	0.025 M
NH_2_	–	–	–	–	–	–	–	–	–
PEG	+	+	+	+	+	+	+	+	+
COOH	+	+	+	+	+	+	+	+	+

(+) stands for excellent dispersibility and stability in time (minimum 1 week), (±) corresponds to colloidal solutions that are stable for <4 days, (−) stands for dispersions which are stable for only a short period of time (between 5 and 10 h). The concentration of nanoparticles in solution was 0.25 mg/mL. The criterion for stability was the absence of visible aggregation or precipitate

If superparamagnetic nanoparticles are used as, for example, an MRI contrast reagent, colloidal stability in (human) serum or plasma is crucial. To prove the value of the presented functionalization method, nanoparticle dispersions in both serum and plasma were prepared and their stability was monitored in time by absorption measurements (see Online Resource 1). Human blood has a pH of 7.4, which will strongly influence the stability of the nanoparticles as was shown in previous paragraphs. Similarly, for the dispersions in buffer solutions, three different coatings were tested, amine, carboxylic acid, and PEG functional groups. These nanoparticles were dispersed in serum and plasma at a concentration of 1 and 0.25 mg/mL. In accordance with the previous results, amine groups on the surface cannot provide sufficient electrostatic repulsion at pH 7.4, which resulted in a rapid decline of the absorbance. On the contrary, carboxylic acid groups and PEG chains should be able to provide sufficient stabilization in these conditions. This was observed in both cases since the absorbance remained constant during the entire experiment, which lasted 48 h. These results indicate that the PEG and carboxylic acid-coated nanoparticles can be of great importance for future in vivo experiments.

An important remark about the use of nanoparticles in biomedical applications is their possible toxicity toward cells. Although many conflicting results were published about the toxicity of superparamagnetic iron oxide nanoparticles, a study by Mahmoudi et al. (2011) recently showed that the surface coating and the cell type itself have a large influence on the possible toxic effects (Zhang et al. 2002). They state that the introduction of a functional surface coating lowers the inherent toxicity. Tartaj et al. (2003) reported that the size, shape, and magnetic dipole moment of the particle also play a role in in vivo experiments. Properties of nanoparticles like longer sedimentation times, higher surfaces areas, and smaller magnetic dipole–dipole interactions might facilitate their use. Nevertheless, a study of the adverse effects toward their environment is necessary for every specific application.

## Conclusions

Oleic acid-coated nanoparticles were functionalized by a reaction with trialkoxy silanes. The reaction takes place in an ultrasonication bath, which reduces the reaction time and prevents crosslinking. The successful coating procedure of the nanoparticles’ surface was proven by FTIR measurements. Multiple techniques (TEM, XRD, VSM) proved the composition of the magnetic core. The obtained functionalized nanoparticles can be dispersed in various aqueous environments including human serum and plasma. Their stability under these conditions was addressed by zeta potential and absorbance measurements, showing a strong relation between the colloidal stability and the pH of the solution. Although PEG-coated nanoparticles also exhibit this dependency, steric hindrance is expected to be more prominent here. In general, the generic method described here allows the introduction of various functional groups on the surface of the nanoparticles. This is particularly useful for subsequent coupling reactions to fluorescent probes, proteins, or substrates. Therefore, we believe that this type of superparamagnetic nanoparticle can be of major importance for future research and applications in biomedicine.

## Electronic supplementary material

Below is the link to the electronic supplementary material.

Supplementary material 1 (PDF 3,794 kb)
