# Supplementary material for: Improved functionalization of oleic acid-coated iron oxide nanoparticles for biomedical applications
Source: J Nanopart Res. 2012 Aug 7;14(9):1100. doi: 10.1007/s11051-012-1100-5 (PMC3460177; doi:10.1007/s11051-012-1100-5)
Supplement: Supplementary file 1 — Supplementary material 1 (PDF 3,794 kb) [file 11051_2012_1100_MOESM1_ESM.pdf]

Improved functionalization of oleic acid-coated iron oxide nanoparticles for biomedical applications

Maarten Bloemen[a]*, Ward Brullot[a], Tai Thien Luong[a], Nick Geukens[b], Ann Gils[c], Thierry Verbiest[a]

[a] *Department of Chemistry, KU Leuven, Celestijnenlaan 200D, box 2425, 3001 Heverlee (Leuven), Belgium*

[b] *PharmAbs, the KU Leuven Antibody Center; KU Leuven, O&N II, Herestraat 49, box 824, 3000 Leuven, Belgium*

[c] *Faculty of Pharmaceutical Sciences, KU Leuven, O&N II Herestraat 49, box 824, 3000 Leuven, Belgium*

0032(0)16327154

maarten.bloemen@fys.kuleuven.be

Supporting information

Protocol human plasma and serum

Human serum and plasma were obtained by the following method: a blood sample from a healthy volunteer was collected in heparinized tubes (BD Vacutainer Systems) and spun at room temperature at a speed of 1300 g for 20 minutes in a swinging bucket centrifuge, with plasma harvested and stored at -20°C until assayed. For serum preparation, a blood sample from the same healthy volunteer was collected in SST™ Serum Separation Tubes (BD Vacutainer Systems), inverted five times and allowed to clot at room temperature for 30 minutes before centrifugation in a swinging bucket centrifuge for 20 minutes at 1300 g; the resultant serum was collected and stored at -20°C until assayed.

Infrared spectra

Following figures show the infrared spectra of the coated nanoparticles. Oleic acid (Figure S1), carboxylic acid (Figure S2), PEG (Figure S3), amine (Figure S4) and thiol (Figure S5) coatings were measured.

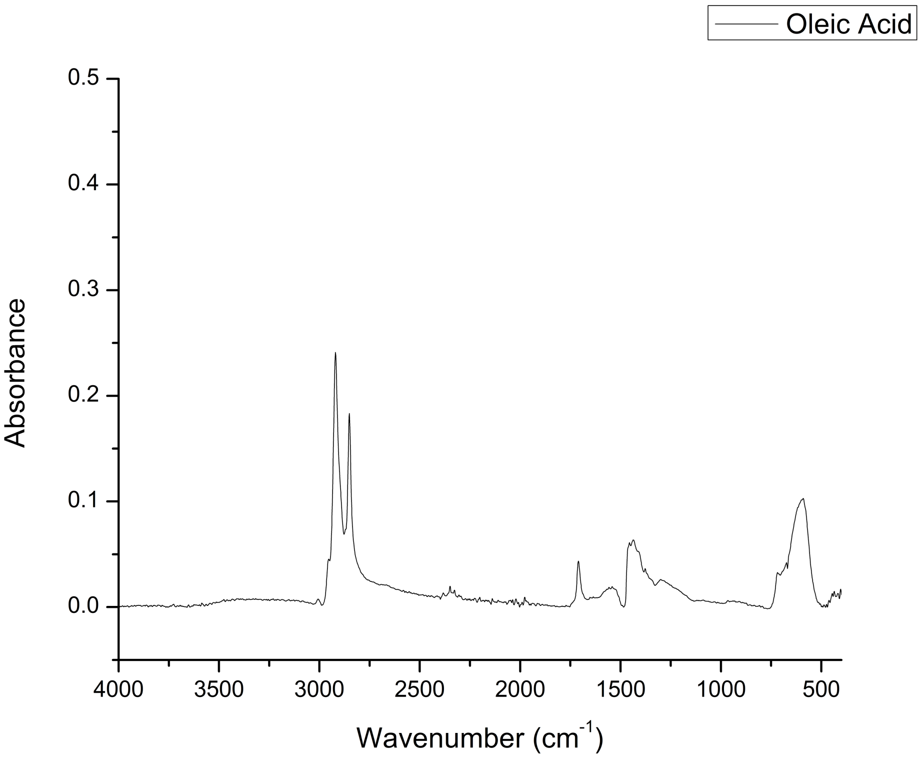

Fig. S1: Infrared spectrum of oleic acid coated nanoparticles.

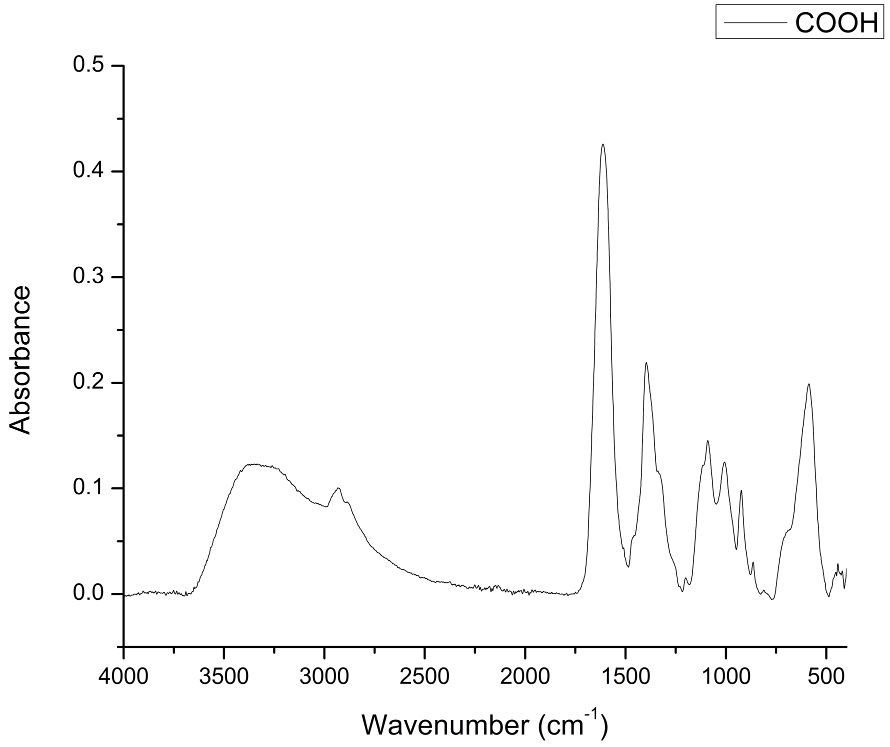

Fig. S2: Infrared spectrum of nanoparticles coated with carboxylic acid groups.

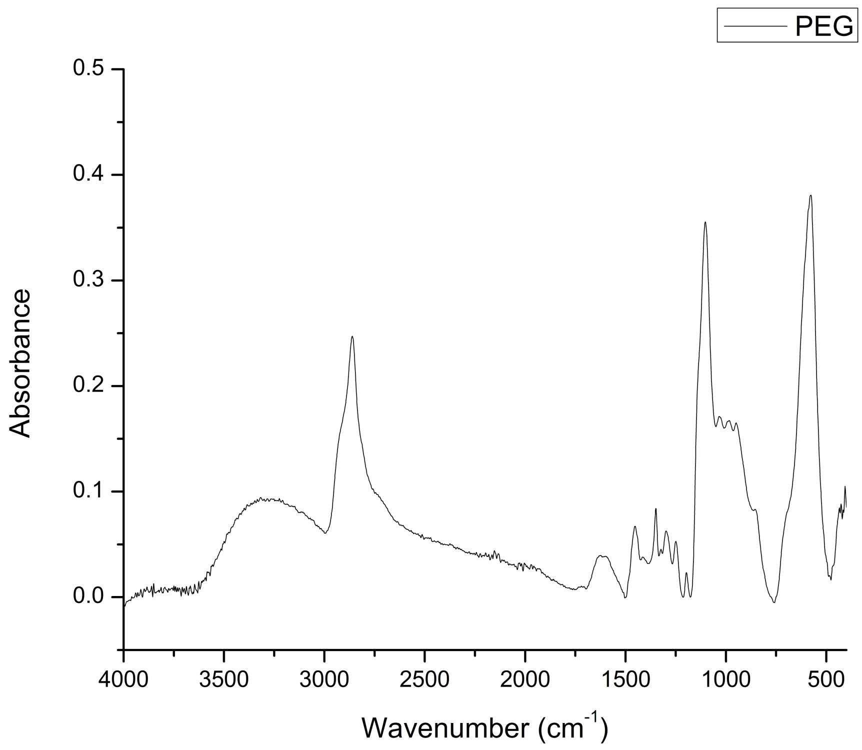

Fig. S3: Infrared spectrum of nanoparticles coated with PEG chains.

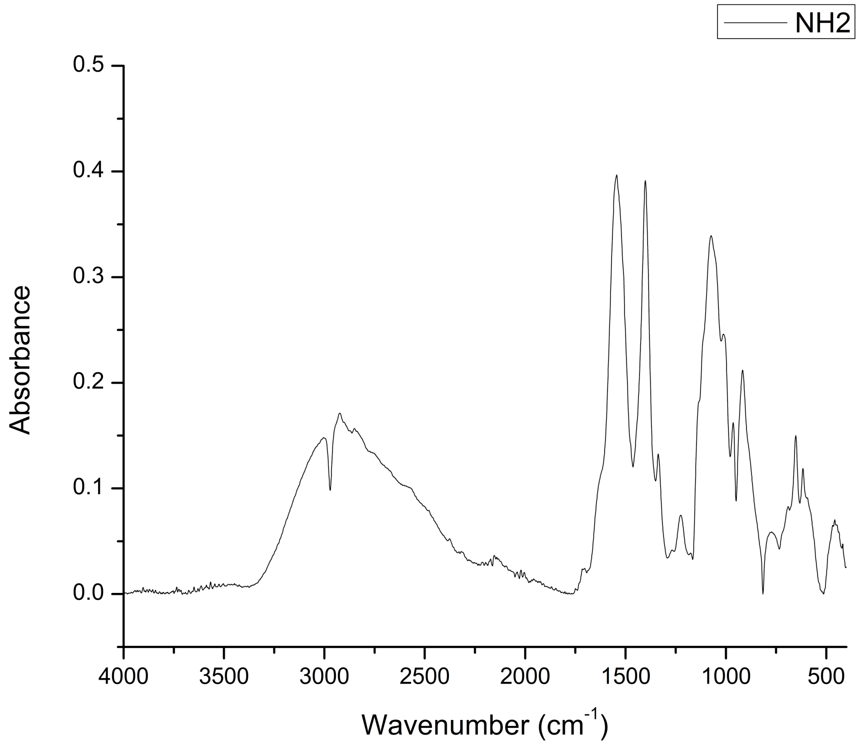

Fig. S4: Infrared spectrum of nanoparticles coated with amine groups.

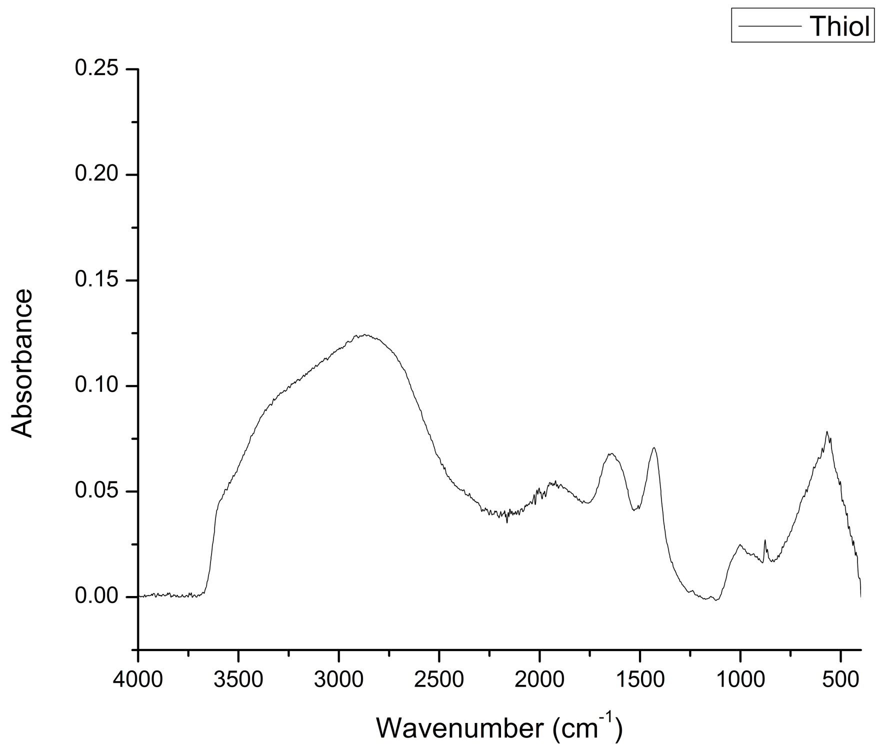

Fig. S5: Infrared spectrum of nanoparticles coated with thiol groups.

Absorbance spectra

The following figures combine the data of the amine (Figure S6), carboxylic acid (Figure S7) and PEG (Figure S8) coated nanoparticles respectively. The concentration of the nanoparticles was 1 or 0.25mg/ml in serum or plasma, as indicated in the legend. Absorbance values were measured at a wavelength of 1000nm.

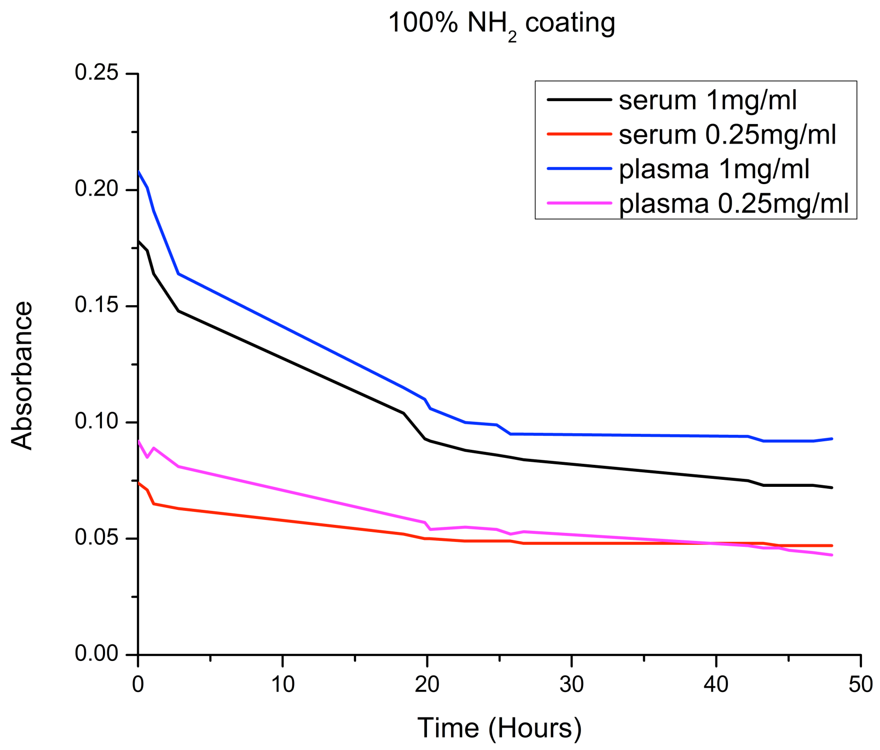

Fig. S6: Absorbance data of the amine coated nanoparticles. The concentration of nanoparticles in serum or plasma was 1 or 0.25mg/ml.

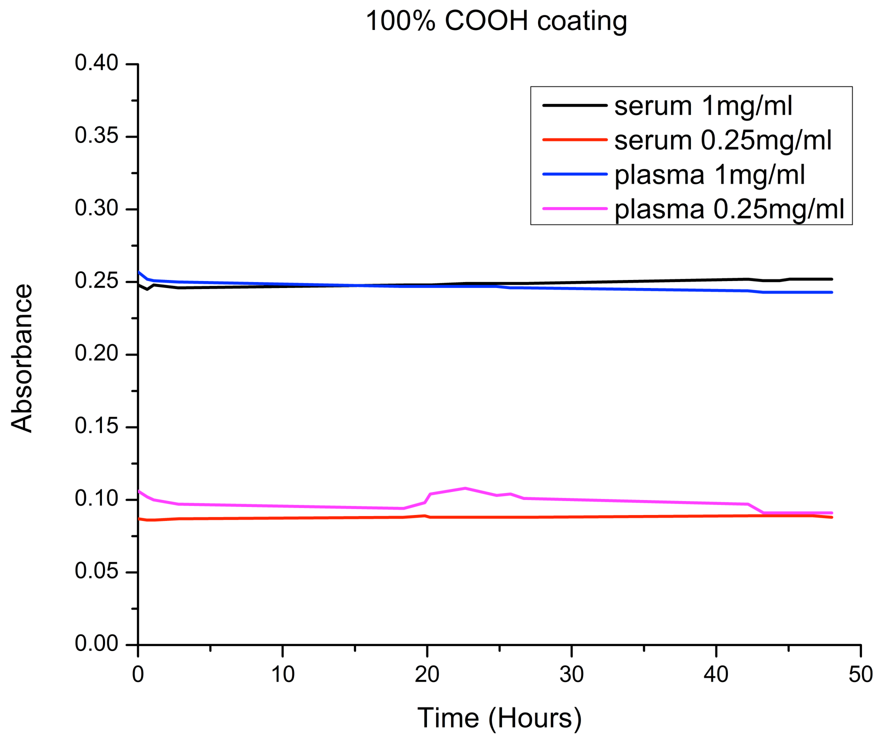

Fig. S7: Absorbance data of the carboxylic acid coated nanoparticles. The concentration of nanoparticles in serum or plasma was 1 or 0.25mg/ml.

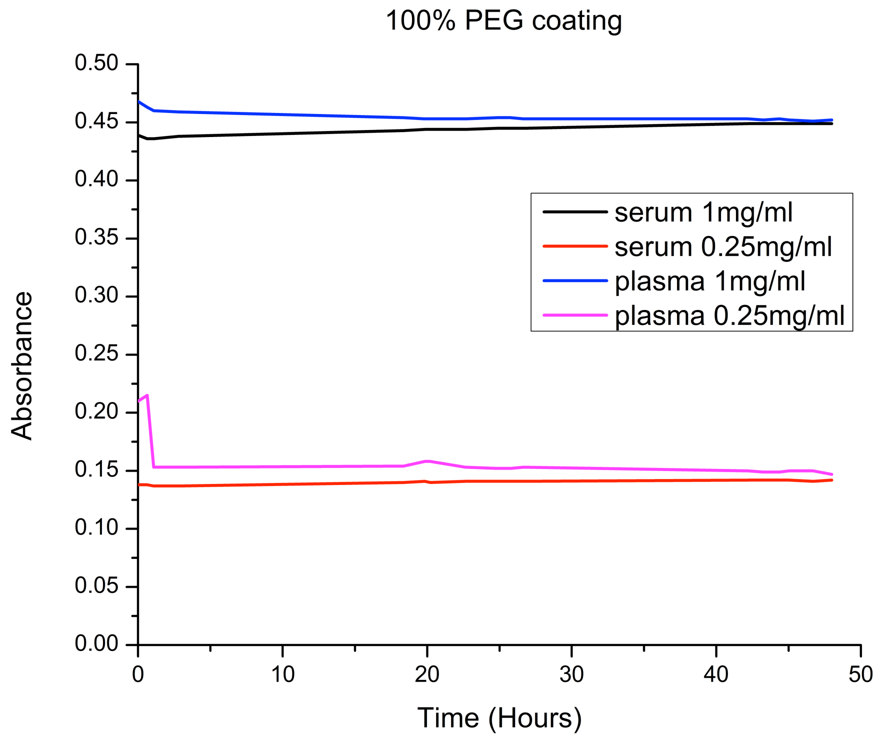

Fig. S6: Absorbance data of the nanoparticles coated with PEG chains. The concentration of nanoparticles in serum or plasma was 1 or 0.25mg/ml.
